# Acipimox inhibits human carbonic anhydrases

**DOI:** 10.1080/14756366.2022.2037579

**Published:** 2022-02-09

**Authors:** Mattia Mori, Claudiu T. Supuran

**Affiliations:** a Department of Biotechnology, Chemistry and Pharmacy, "Department of Excellence 2018-2022", University of Siena, Siena, Italy; b Neurofarba Department, Pharmaceutical and Nutraceutical Section, University of Florence, Sesto Fiorentino, Italy

**Keywords:** Carbonic anhydrase, acipimox, docking, molecular dynamics, lipolysis

## Abstract

Acipimox, a nicotinic acid derivative in clinical use for the treatment of hyperlipidaemia, incorporates a free carboxylic acid and an N-oxide moiety, functionalities known to interact with the metalloenzyme carbonic anhydrase (CA, EC 4.2.1.1) and inhibit its activity. Herein we report that acipimox acts as a low micromolar CA inhibitor (CAI) against most human (h) isoforms possessing catalytic activity, hCA I – XIV. By using computational techniques (docking and molecular dynamics simulations), we propose that acipimox coordinates through its carboxylate group to the zinc ion from the enzyme active site cavity, whereas the N-oxide group is hydrogen-bonded to the proton shuttle His residue in some isoforms (hCA I) or to active site Thr or Gln residues in other isoforms (hCA II, III, IV, VII, etc). As some CA isoforms are involved in lipogenesis, these data may be useful for the design of more effective CAIs with antiobesity activity.

## 1. Introduction

Acipimox (Olbetam^R^) **1** is a clinically used drug for the treatment of hyperlipidaemic patients that do not respond to other therapeutic regimens^1^. The drug exerts its hypolipidemic effect by inhibiting lipolysis as well as the free fatty acid flux to the liver, by reducing the precursor pool size of very low density lipoprotein (VLDL)-triglyceride and inhibiting VLDL synthesis, with the consequent reduction of plasma triglyceride levels and increase of high density lipoprotein (HDL) cholesterol^2^. The drug also interferes with peroxisomal oxidative activities and enhances autophagy, and for such reasons it was proposed as one of the first therapeutic agents for healthy ageing by Bergamini’s group^3^. It is known that autophagy plays a crucial role in cell housekeeping processes during fasting, contributing to the removal of altered membranes, mitochondria, as well as other organelles, including peroxisomes, which may explain the antiaging effects of caloric restriction, which in turn can be enhanced by using anti-lipolytic drugs such as acipimox at dosages lower than those used for the treatment of hyperlipidaemia^4^.

Carboxylates constitute one of the most versatile classes of carbonic anhydrase (CA, EC 4.2.1.1) inhibitors (CAIs)^5–7^. Kinetic, crystallographic, computational and other biophysical studies evidenced a multitude of possibilities by which carboxylates inhibit these enzymes, among which direct coordination to the metal ion, anchoring to the metal ion-coordinated water molecule, occlusion of the active site entrance or even binding outside the active site cavity in a hydrophobic pocket adjacent to the entrance to the catalytic site^8–10^. Various classes of aliphatic, aromatic, and heterocyclic carboxylates were investigated in detail as CAIs in the last decades^5–10^ and many such compounds showed significant inhibitory action and selectivity against the many catalytically active CA isoforms (of the 15 presently known), some of which are involved in crucial physiological or pathological phenomena^11^. Although no carboxylate CAI is in clinical use at this moment, sulphonamides, sulfamates or sulfamides with such an action are widely employed for the treatment of many diseases, among which edoema, glaucoma, epilepsy, obesity, hypoxic tumours, etc.^8–12^, whereas other such compounds are under investigation for the management of cerebral ischaemia, neuropathic pain or arthritis^13^. Recently, we have examined carboxylate **2**^6^, which is structurally similar to acipimox **1** as a CAI, finding that it acts as a weak inhibitor of several human isoforms. We thus decided to investigate whether acipimox possesses such an activity and investigated in detail its interaction with all catalytically active human isoforms (hCA I – XIV) by means of kinetic and computational studies.

## 2. Materials and methods

### 2.1. Computational studies

The crystallographic structures of hCAs available in the Protein Data Bank (PDB) were retrieved under the following accession codes, and used as rigid receptors in molecular docking simulations: hCA I (PDB-ID: 6I0J)^14^, hCA II (PDB-ID: 3K34)^15^, hCA III (PDB-ID: 2HFW)^16^, hCA IV (PDB-ID: 3FW3)^17^, hCA VI (PDB-ID: 3FE4)^18^, hCA VII (PDB-ID: 6H38)^19^, hCA IX (PDB-ID: 5FL4)^20^, hCA XII (PDB-ID: 1JD0)^21^, hCA XIII (PDB-ID: 3CZV)^22^, hCA XIV (5CJF)^23^. For hCAs structures in complex with covalent Zn(II) binders, the Zn(II)-bound water molecule was modelled as described previously^7^. Atomic RESP charges of acipimox were computed at the HF/6-311G* level of theory using Gaussian 16 rev. C0.01^24^. Molecular docking was carried out with the GOLD program (The Cambridge Crystallographic Data Centre) version 2020^25^^,^^26^, by centreing a spherical binding site having a radius of 14 Å on the catalytic Zn(II) ion. During docking, the Zn(II)-bound water molecule was handled by the “toggle” function of the GOLD program. The CHEMPLP docking function was used for docking and scoring purposes. Docking complexes between acipimox and hCA IX were relaxed through molecular dynamics (MD) simulations carried out with AMBER18^27^. The ff14SB force field was used to parametrise the protein, while the ligand was parametrised with the General Amber Force Field (GAFF) using the RESP partial atomic charges^28^^,^^29^. Each complex was solvated in a rectangular box of TIP3P-type water molecules buffering 6 Å from the protein. Na^+^ counter-ions were added to neutralise the total charge of the system. The bound approach was used to parametrise the catalytic Zn(II) ion, to which an arbitrary charge of +1 was assigned based on a previous work^7^. The protocol already described elsewhere was used to run MD simulations^30–33^. In brief, the solvent was first energy minimised for 500 steps using the steepest descent algorithm (SD) followed by 2500 steps with the conjugate gradient algorithm (CG) while keeping the solute as frozen. The solvated solute was then energy minimised for 1000 steps with the SD and subsequent 5000 steps with the CG before heating to 300 K for 1 ns using the Langevin thermostat. The system’s density was equilibrated for 1 ns by the Berendsen barostat, then the system was preliminarily equilibrated for 50 ns before running the final production of MD trajectories for 150 ns. A time-step of 2 fs was used. Three independent MD replicas were run for each acipimox/hCA IX docking complex. Analysis of MD trajectories was carried out with the CPPTRAJ software^34^.

### 2.2. Carbonic anhydrase inhibition

The CA-catalysed CO_2_ hydration activity has been measured with an Applied Photophysics stopped-flow instrument^35^. The used pH indicator was phenol red (at a concentration of 0.2 mM), working at the absorbance maximum of 557 nm. 10 mM HEPES (pH 7.4) was employed as a buffer, in the presence of 10 mM NaClO_4_ to maintain the ionic strength constant. The initial rates of the CA-catalyzed CO_2_ hydration reaction were followd up for a period of 10 to 100 s. The substrate CO_2_ concentrations ranged from 1.7 to 17 mM for determining the inhibition constants. For each inhibitor, at least six traces of the initial 5–10% of the reaction were used to determine the initial velocity. The uncatalyzed rates were determined in the same manner and subtracted from the total observed rates. Stock solutions of inhibitors (10 mM) were prepared in distilled-deionized water with maximul 5% DMSO, and dilutions up to 10 nM were done thereafter with the assay buffer. Inhibitor and enzyme solutions were preincubated together for 15 min prior to the assay, in order to allow for the formation of the E-I complex. The inhibition constants were obtained by non-linear least-squares methods using Prism 3 and the Cheng-Prusoff equation, as reported previously^36–38^, and represent the mean from at least three different determinations. The hCAs concentration in the assay system were of 5.3–15.8 nM. All human enzymes were recombinant proteins obtained in-house, as described earlier^36–38^.

### 2.3. Chemicals

Acipimox, acetazolamide, buffers and other reagents were the highest purity compounds available from Sigma-Aldrich (Milan, Italy).

## 3. Results

### 3.1. CA inhibition

Acipimox **1** was tested for the inhibition of all catalytically active human CA isoforms, hCA I – XIV by using a stopped-flow CO_2_ hydrase assay^35^ (Table 1). Compound **2**, structurally related to acipimox, and acetazolamide **3**, a standard sulphonamide CAI were also included in Table 1 for comparison reason, as their inhibition profile was determined in the same manner as for **1**^6^.

**Table 1.** hCA I-XIV inhibition data with compounds **1–3** by a stopped-flow CO_2_ hydrase assay^35^.

	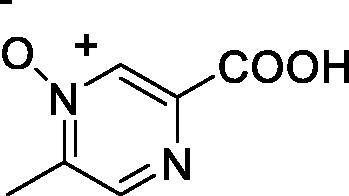	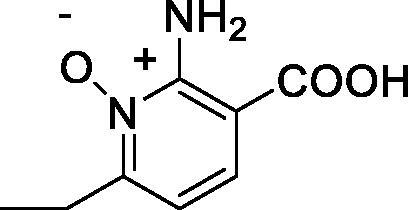	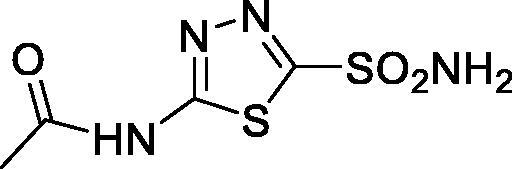
K_I_* (µM)	**1**	**2** ^#^	**3**
hCA I	3.3	72	0.25
hCA II	7.5	>100	0.012
hCA III	5.4	>100	20
hCA IV	8.8	>100	0.074
hCA VA	7.5	NT	0.063
hCA VB	2.8	NT	0.054
hCA VI	7.0	NT	0.011
hCA VII	9.2	NT	0.002
hCA IX	6.3	NT	0.025
hCA XII	8.4	NT	0.006
hCA XIII	87	NT	0.017
hCA XIV	5.9	NT	0.041

*Mean from 3 different assays, by a stopped flow technique (errors were in the range of ± 5–10% of the reported values, data not shown). NT: not tested.

^#^From ref. [6].

Data from Table 1 show that acipimox is an effective micromolar inhibitor of all CA isoforms (K_I_s in the range of 2.8–9.2 µM) except hCA XIII for which the inhibition constant is an order of magnitude higher, i.e. 87 µM. The isoforms which were more effectively inhibited by acipimox were hCA VB and hCA I, with K_I_s of 2.8 and 3.3 µM, respectively. Although the structurally related compound **2** was tested only on a limited number of isoforms, it seems to be much less effective compared to acipimox as a CAI (Table 1). On the other hand, the sufonamide inhibitor **3** is highly effective against all isoforms except hCA III, but this is a well understood phenomenon which has been discussed in details elsewhere^39^.

### 3.2. Computational studies

**Figure 1.**
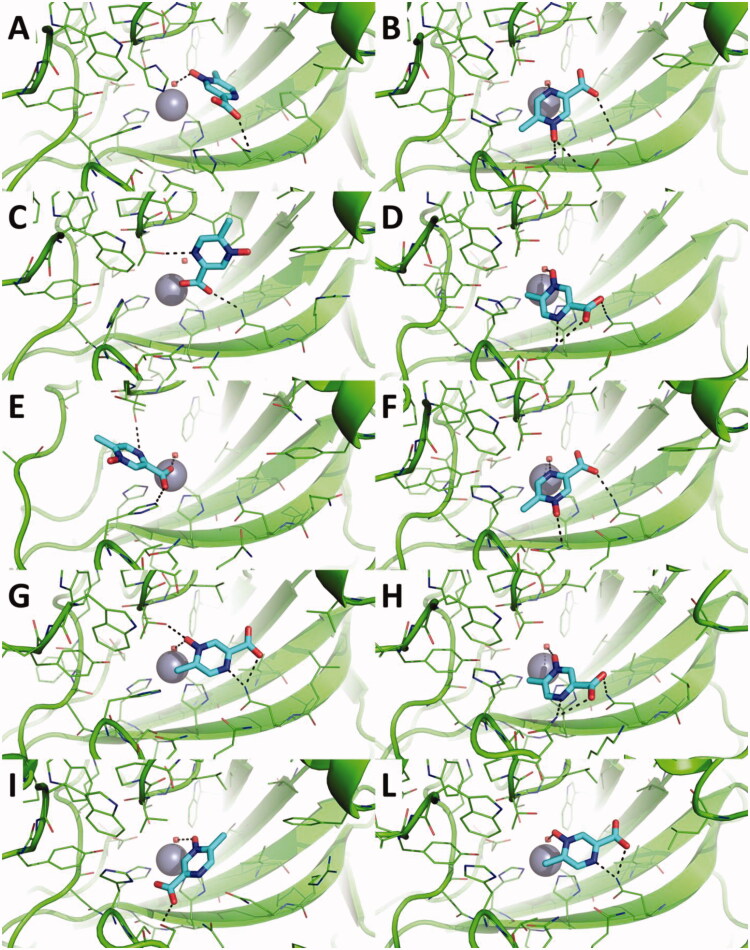
Predicted binding mode of acipimox against the crystallographic structure of hCAs in which the Zn(II)-bound water molecule was considered explicitly during docking. A) hCA I; B) hCA II; C) hCA III; D) hCA IV; E) hCA VI; F) hCA VII; G) hCA IX; H) hCA XII; I) hCA XIII; L) hCA XIV. hCAs are shown as green cartoon, residues within 5 Å from acipimox are shown as green lines. Acipimox is shown as cyan sticks, the catalytic Zn(II) ion as a grey sphere, and the Zn(II)-coordinated water molecule as a small red sphere. Polar interactions are highlighted by black dashed lines.

**Figure 2.**
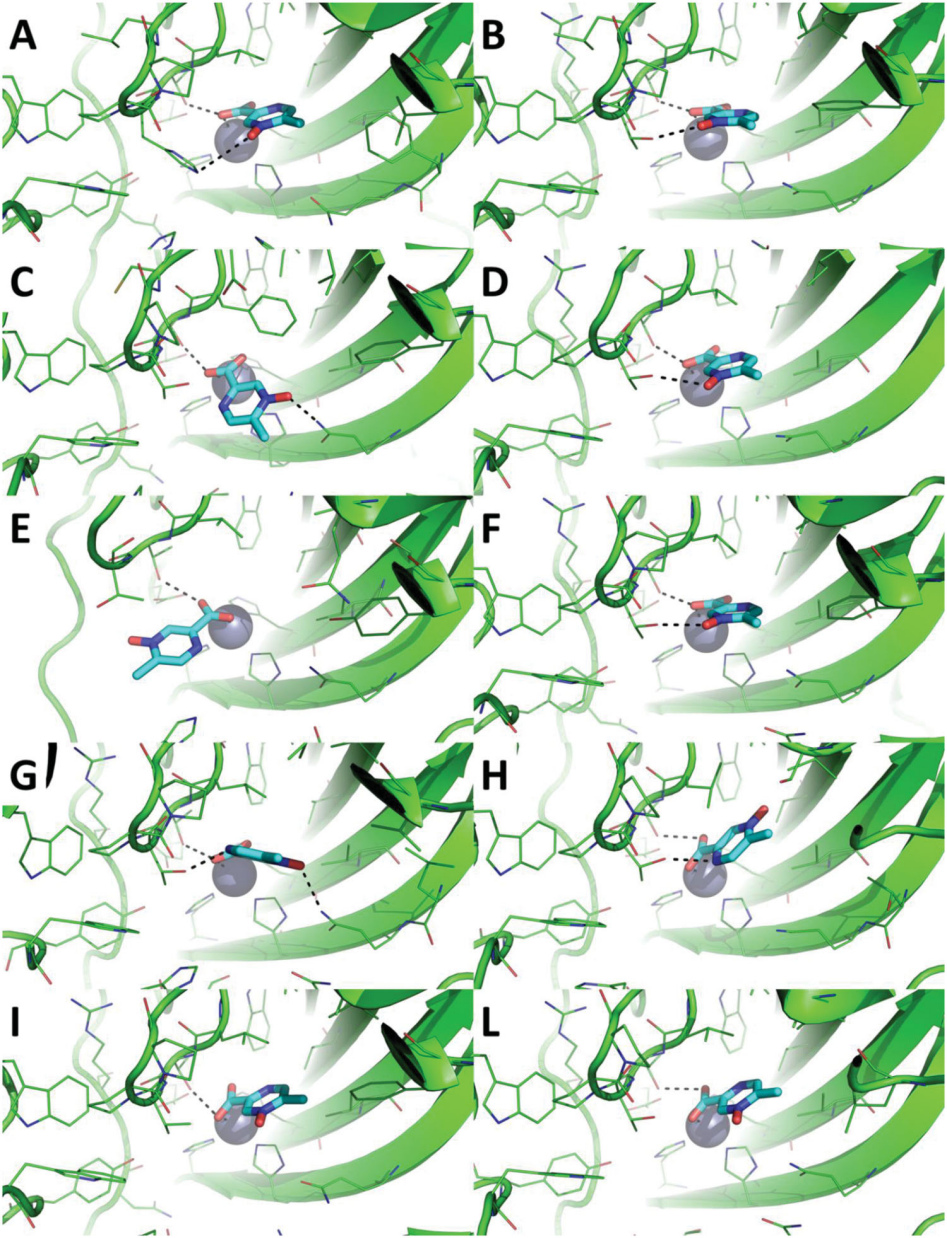
Predicted binding mode of acipimox against the crystallographic structure of hCAs in which the Zn(II)-bound water molecule was not considered in docking simulations. A) hCA I; B) hCA II; C) hCA III; D) hCA IV; E) hCA VI; F) hCA VII; G) hCA IX; H) hCA XII; I) hCA XIII; L) hCA XIV. hCAs are shown as green cartoon, residues within 5 Å from acipimox are shown as green lines. Acipimox is shown as cyan sticks, the catalytic Zn(II) ion as a grey sphere. Polar interactions are highlighted by black dashed lines.

The possible interaction between acipimox and hCAs was investigated by molecular docking and MD simulations. While docking is a highly powerful tool to rapidly predict the possible ligand binding mode and score towards a macromolecular target, MD simulations are more time-demanding calculations that explore the conformational and energy evolution of a molecular system in physiological conditions over the time^40^. First, the RESP atomic partial charges of acipimox were computed at the HF/6-311G* level of theory, and used in subsequent molecular simulations. Molecular docking was carried out against hCAs investigated in this work, for which the structure has been elucidated preferentially by X-ray crystallography and NMR spectroscopy, and is available in public repositories such as for instance the PDB^41^. This choice did not allow to predict the binding mode of acipimox against hCA VA and VB isoforms. Given the multiple binding modes of acipimox pharmacophores to hCAs, as underlined in several recent reviews and works^7^ here we carried out docking simulations by considering the Zn(II)-bound water molecule as explicit or by removing it. As expected, the binding mode and score of acipimox is significantly different in the two scenarios. In docking to hCAs having the Zn(II)-bound water molecule (Figure 1), acipimox mostly interacts with the water molecule by H-bond interactions established by the N-oxide group. This interaction pattern was observed in the docking pose of acipimox against hCA I, hCA IV, hCA IX, and hCA XII-XIV (Figure 1(A,D,G–L)). In these docking poses, the carboxylic group projects towards the entrance of the catalytic site, where it interacts with Asn, Gln or Ser residues also thanks to the possible cooperation of the pyrazine N atom. Docking against hCA II and hCA VII shows the aromatic N atom binding to the Zn(II)-bound water molecule (Figure 1(B,F)), while only in the case of hCA VII the water molecule is anchored by the carboxylic group (Figure 1(E)). Docking against hCA III shows a peculiar binding mode, i.e. acipimox is unable to bind the water molecules but seems to occlude the catalytic site of the enzyme (Figure 1(C)). Overall, when the water molecule coordinated to the catalytic Zn(II) ion is considered explicitly, acipimox can potentially adopt different binding modes within the highly conserved active site of hCAs. In contrast, when docking simulations were carried against hCAs that do not bear the water molecule within the Zn(II) coordination system, a very consistent binding mode of acipimox was obtained (Figure 2). Specifically, the catalytic Zn(II) ion is coordinated by the carboxylate group, which also contacts a Thr residue by H-bond interactions. The N-oxide group is H-bonded to the proton shuttle His residue in hCA I (Figure 2(A–I)) and to Thr or Gln residues in other isoforms but hCA VI and hCA XII-XIV. Only in hCA IX and hCA XII, the pyrazine N atom is H-bonded to a Thr residue (Figure 2(G–L)). Notably, comparison of docking scores sharply highlights the tighter binding of acipimox to the catalytic Zn(II) ion than the Zn(II)-coordinated water molecule (Table 2), also showing a homogeneous distribution of theoretical affinity values that is in agreement with experimental results.

**Table 2.** GOLD CHEMPLP scores of acipimox against a panel of hCAs.

	CHEMPLP score	
	Zn(II)-bound water molecule	Catalytic Zn(II) ion
hCA I	37.45	53.12
hCA II	49.08	56.10
hCA III	41.23	53.74
hCA IV	44.37	52.31
hCA VI	47.85	51.76
hCA VII	43.13	55.62
hCA IX	46.43	54.73
hCA XII	48.90	59.75
hCA XIII	46.61	53.17
hCA XIV	46.36	55.83

Overall, docking simulations suggest that acipimox is able to fit the active site of a panel of hCAs, and that it binds preferentially to the catalytic Zn(II) ion than the Zn(II)-coordinated water molecule.

**Figure 3.**
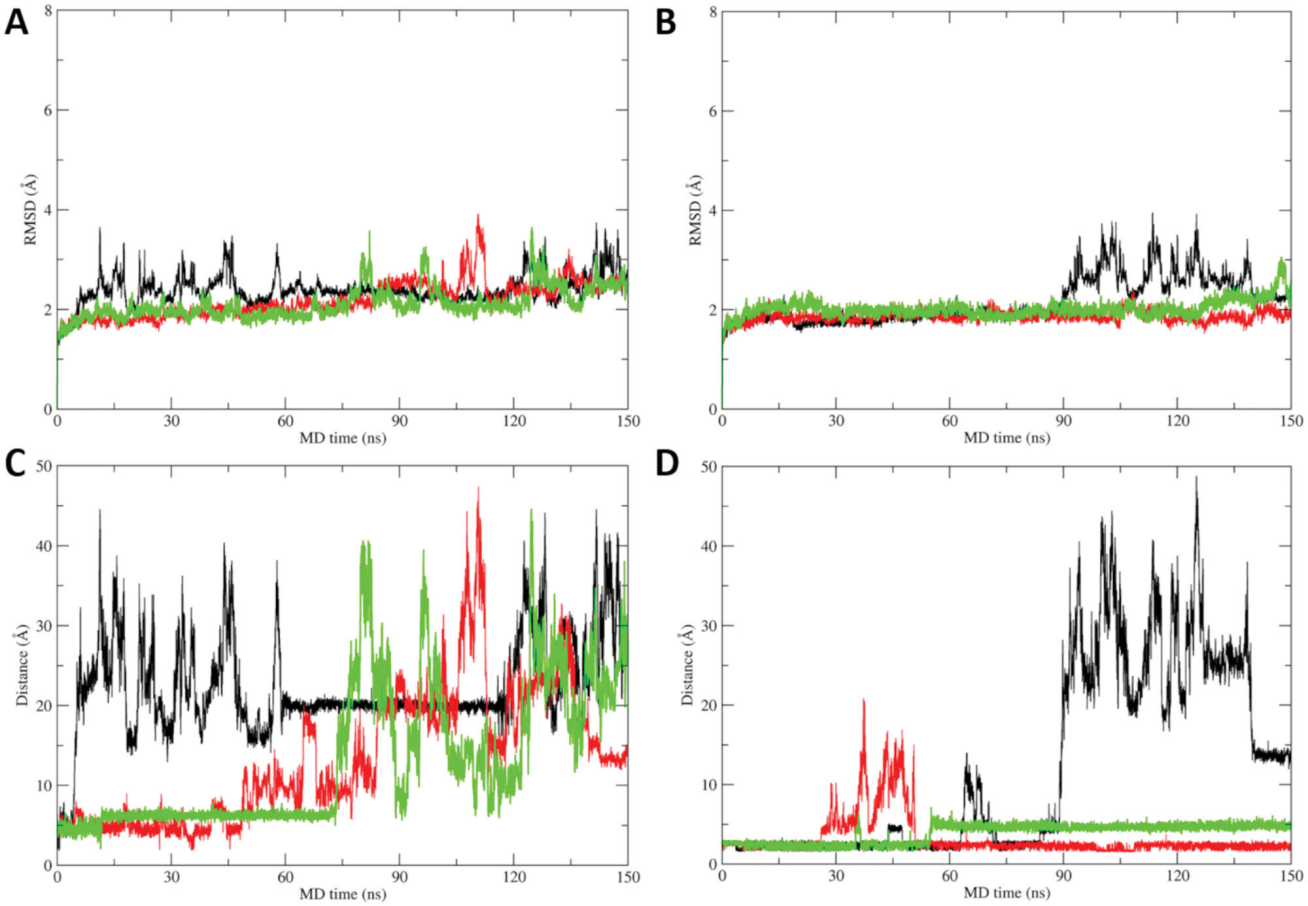
A) RMSD plot of hCA IX active site residues and acipimox along MD simulation time in each replica carried out from docking poses H-bonded to the Zn(II)-bound water molecule. B) RMSD plot of CA IX active site residues and acipimox along MD simulation time in each replica carried out from docking poses in which acipimox is directly bound to the catalytic Zn(II) ion. C) Plot of the distance between the N-oxide group of acipimox and the catalytic Zn(II) ion of hCA IX along MD time. D) Plot of the distance between the carboxylic group of acipimox and the catalytic Zn(II) ion of hCA IX along MD time. Each replica is shown with different colour (i.e. black, red, green).

To further corroborate the binding hypothesis emerged by docking analysis, MD simulations were carried out on docking complexes between acipimox and hCA IX, which was selected as a case study due to its role as a drug target in anticancer therapy^10^^,^^11^. Three replicas of 150 ns of MD simulations were run, for a total simulation time of 450 ns on each acipimox/hCA IX complex. Results clearly show that the interaction between acipimox and the Zn(II) bound water molecule is unstable in every replica, as underlined by the analysis of the root mean square deviation (RMSD) of active site residues and acipimox, as well as by the distance plots between the zinc-binding group of acipimox in the two poses and the catalytic Zn(II) ion (Figure 3). In fact, starting from the docking pose in which acipimox is H-bonded to the Zn(II)-coordinated water molecule (Figure 1(G)), in every replica the compound detaches from the active site of the enzyme and moves into the bulk solvent (Figure 3(A,C)). In a single replica (see black line in Figure 3(C)), acipimox interacts in a site of CA IX that was already characterised in hCAs for the interaction of small molecules^10^^,^^11^, although this is a transient interaction in MD simulations.

**Figure 4.**
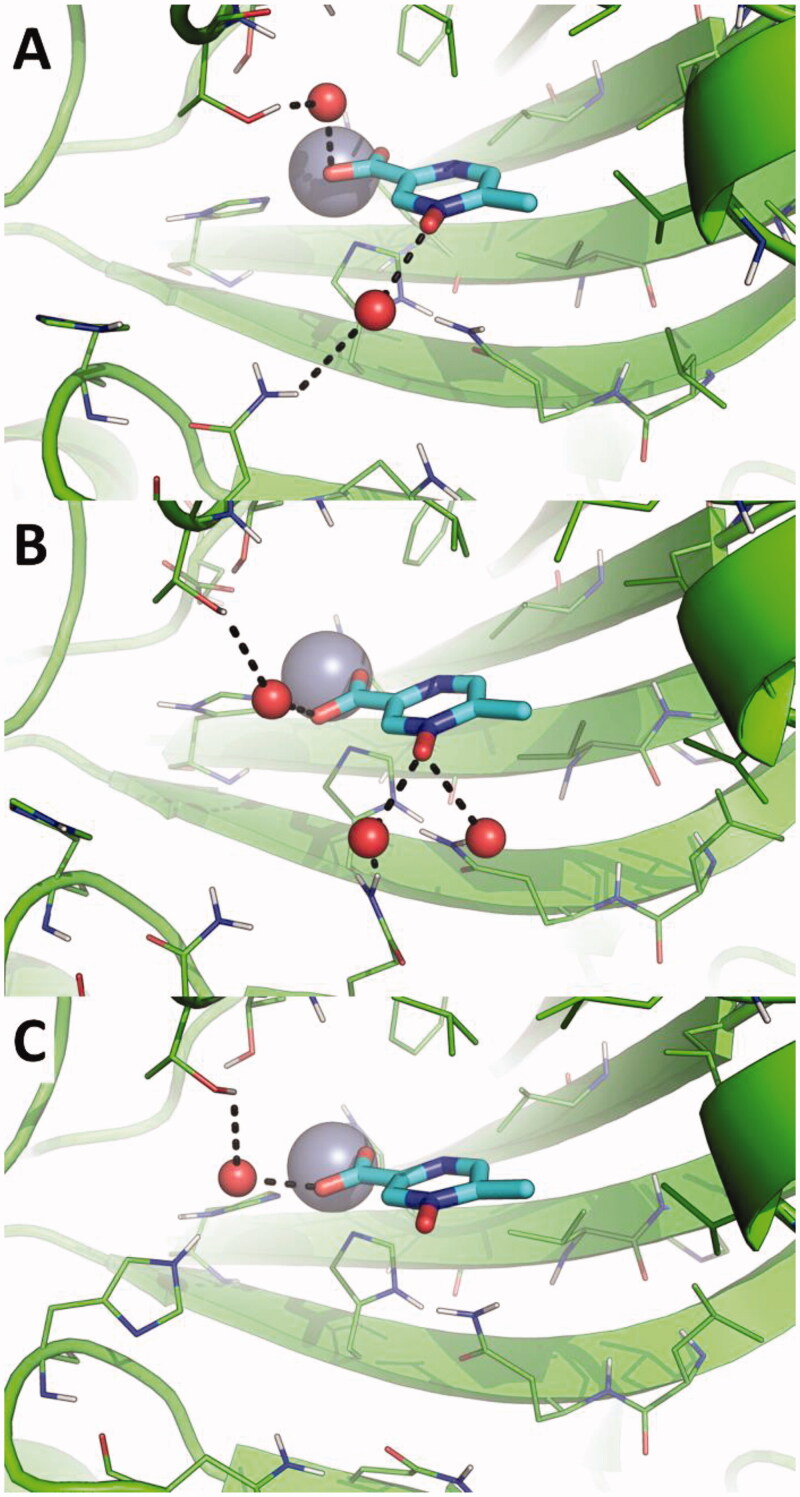
Magnification of the binding mode of acipimox within the catalytic site of hCA IX in the representative frames extracted by cluster analysis from three independent MD replicas. hCA IX is shown as green cartoon, residues within 5 Å from acipimox are shown as green lines. The catalytic Zn(II) ion is shown as a grey sphere, while bridging water molecules are shown as small red spheres. The bulk solvent and non-polar H atoms were removed. Polar interactions are highlighted as black dashed lines.

In contrast, the direct binding of acipimox to the catalytic Zn(II) ion is much more stable and persistent in time, as observed in two MD replicas (Figure 3(D), red and green line). In one of these latter, a conformational switch of acipimox is observed (Figure 3(D), green line), whereas in the third replica acipimox is extruded from the hCA IX active site after around 90 ns of MD simulations (Figure 3(D), black line). A representative MD frame was extracted from each MD replica by cluster analysis carried out on acipimox and residues of the hCA IX active site. Visual inspection of representative frames clearly highlights a highly consistent binding mode of acipimox in the three replicas (Figure 4). The compound interacts with the catalytic Zn(II) ion and with Thr201 through a water-bridged H-bond, while additional water-bridged interactions are established with Gln residues near the entrance of the active site (Figure 4(A,B)).

## 4. Conclusions

We demonstrate here that acipimox, a clinically used drug for the treatment of hyperlipidaemia, is also an effective, micromolar inhibitor of all human CA isoforms except hCA XIII for which the inhibitory effects were weak. Being a carboxylic acid derivative, and as we were unable to resolve the X-ray crystal structure of an adduct of any CA isoform with this drug, we employed computational techniques for understanding its inhibition mechanism. Docking results were corroborated by MD simulations, clearly showing that acipimox preferentially binds directly the catalytic Zn(II) ion of hCAs in a monodentate fashion with an oxygen atom from the carboxylate zinc-binding group. Furthermore, the binding of the drug to the Zn(II)-coordinated water molecule was shown to be less stable and persistent in time. The interactions established by acipimox within the hCAs active site are nicely overlapping with those observed for compounds having similar pharmacophores (COO^-^ as zinc-binding group), which may thus shed further light on the ability of this small molecular drug to inhibit hCAs. As acipimox interferes with the lipid metabolism, and since some CAIs are used for the treatment and prevention of obesity^12^, these data may be useful for the design of more effective carboxylate CAIs with pharmacological applications.
